# Heart Failure in Black Populations: Epidemiology, Pathophysiology, and Treatment Disparities

**DOI:** 10.1007/s40615-025-02371-3

**Published:** 2025-03-07

**Authors:** Kazi N. Islam, Rahib K. Islam, Ivan Nguyen, Yasmeen Magherahabed, Humza Pirzadah, M. Bazlur Rashid

**Affiliations:** 1 Central State University, Wilberforce, OH 45384 USA; 2 School of Medicine, Louisiana State University Health Sciences Center, New Orleans, LA 70112 USA; 3 University of Houston-Clear Lake, 2700 Bay Area Blvd, Houston, TX USA; 4 Department of Agricultural Research Development Program, Central State University, 1400 Brush Row Road, Wilberforce, OH 45384 USA

**Keywords:** Heart failure, Black population, HF

## Abstract

Heart failure (HF) remains a significant health challenge globally, placing a heavy burden on individuals, families, and healthcare systems. The prevalence of HF continues to rise, posing substantial public health concerns. This burden is particularly pronounced among the Black Population, who face higher prevalence, earlier onset, and greater severity of HF compared to other racial and ethnic groups. This review explores the multifaceted landscape of HF in Black individuals by examining epidemiological patterns, pathophysiological mechanisms, clinical presentations, treatment disparities, and clinical outcomes. Black individuals exhibit distinct pathophysiological characteristics, such as genetic variations contributing to heightened susceptibility and severity of HF. Social determinants of health, including socioeconomic status, education, and healthcare access, further exacerbate these disparities. Despite advancements in medical science, Black individuals receive less optimal HF care, reflected in lower rates of guideline-directed medical therapy and cardiac rehabilitation. Addressing these disparities requires targeted interventions and a holistic approach that emphasizes social determinants of health, improved healthcare access, and health equity. This review synthesizes existing literature to illuminate the unique challenges faced by Black HF patients and advocates for evidence-based strategies to enhance management and outcomes, aiming to reduce disparities and improve the well-being of this vulnerable population.

## Introduction

While HF remains a global health challenge affecting millions, this review emphasizes the racial disparities observed among Black individuals in the USA. Black individuals face higher prevalence, earlier onset, and greater severity of HF compared to other racial and ethnic groups, warranting a focused examination of epidemiological patterns, clinical presentations, and treatment disparities within this demographic [1].

The notable imbalance in HF prevalence among Black individuals highlights the crucial need for customized interventions and a thorough examination of the underlying factors driving this disparity [2]. Despite advances in medical science and healthcare delivery, Black individuals continue to experience a higher prevalence, earlier onset, and increased severity of HF compared to other racial and ethnic groups [3].

This review aims to delve into the multifaceted landscape of HF in Black individuals, unraveling the intricate connection of epidemiological patterns, pathophysiological mechanisms, clinical presentations, treatment disparities, and clinical outcomes. By synthesizing existing literature, we aim to illuminate the unique challenges faced by Black patients faced with HF and explore evidence-based interventions and strategies aimed at mitigating these disparities.

HF remains a pressing healthcare challenge worldwide, placing significant strain on individuals, families, and public health systems. Although it affects millions globally, the burden of HF is particularly severe among Black in the USA, who experience higher prevalence, earlier onset, and greater severity of the condition. These disparities stem from distinct pathophysiological factors, such as genetic variations, social determinants of health, and differences in healthcare access and quality. Black population is more likely to present with comorbidities such as hypertension and diabetes, which exacerbate the risk and severity of HF. Despite advancements in medical care, Black often receives less optimal HF management, including lower rates of guideline-directed medical therapy and limited access to cardiac rehabilitation services. To address these disparities, targeted interventions are necessary, focusing on improving healthcare access, addressing social determinants, and promoting health equity.

In synthesizing these insights, we aim to develop a comprehensive understanding of the condition of HF and several issues surrounding it in Black individuals, bringing forth meaningful dialogue and actionable initiatives to ameliorate disparities and enhance the well-being of vulnerable populations.

It is important to acknowledge that race is a social construct rather than a biological determinant. The racial categorizations used in this review, such as “Black” reflect complex social, historical, and economic factors that influence health disparities. These categories are used to examine inequities in healthcare access and outcomes. Still, it is crucial to recognize that the observed differences in health are largely due to social determinants of health, structural racism, and systemic inequalities.

This review uses the term “Black” to refer specifically to individuals of Black descent born or residing in the USA. This term (Black) is also used more broadly to include individuals of Black descent globally, including Black populations, as well as Black immigrants and other individuals belonging to the Black racial group.

## Epidemiology of HF in Black Individuals

HF in the USA affects 6.7 million people [4]. Throughout a lifetime, it is estimated that 1 in 4 people will suffer from HF, and as a result, the total number of HF patients is forecasted to increase to a prevalence of roughly 8.5 million people in 2030 [5]. Likewise, the total predicted costs for treating HF will amount to $70 billion in 2030 [6]. The increasing volume of HF cases and the looming burden of treatment costs highlight the importance of prevention and the search for a proactive strategy to decrease the incidence of HF. Investigating risk factors is an essential step in mitigating the rising number of cases.

One of the most evident factors for HF is race, and more specifically, it was found that Black individuals had a higher prevalence of HF, more hospitalizations, and a higher mortality rate from HF when compared to Asian, White, and Hispanic individuals [4, 6]. The incidence of HF in Black individuals was 4.6 per 1000 person-years, while the incidence for non-Hispanic White individuals was lower at 2.4 per 1000 person-years. It is interesting to note that the lowest incidence rate for HF was in Chinese Americans at 1 per 1000 person-years [1]. Thus, in 2030, it is expected that the prevalence of HF in Black individuals will be as high as 3.6% compared to 3.2% in non-Hispanic White individuals [5]. As for HF-related mortalities, the mortality rate has been steadily increasing, especially after COVID-19, as of 2024, despite its decreased rate between 1999 and 2012 [7, 8]. The age-adjusted HF mortality rate in Black individuals was higher than in non-Hispanic White individuals at 41.4/100,000 compared to 33.1/100,000, respectively. Furthermore, not only did Black populations have the highest all-cause mortality from HF, but the mortality rate for both Black men and women below 65 years of age showed the highest increase in rate between 2010 and 2020 [5]. The total number of hospitalizations related to HF was highest among Black patients, and Black patients accounted for around 50% of the HF hospitalizations for patient ages between 18 and 45 [5]. The statistics evaluating race and HF are deeply concerning. They reveal the disparity in our healthcare system and emphasize the need to examine the factors primarily responsible for differences in HF prevalence and outcomes.

Some of the most common risk factors for developing HF include hypertension, smoking, diabetes mellitus, obesity, and atherosclerosis [9, 10]. When the risk factors of hypertension, diabetes, and obesity were analyzed in adults over the age of 20, there was a higher prevalence of all diseases in Black individuals when compared to Hispanic, Asian, and White Americans [11]. The higher prevalence of risk factors contributes to the higher incidence of HF in Black individuals [12].

Social determinants of health have also been extensively studied and used to predict the incidence and outcomes of many different disease courses. These social determinants can include socioeconomic status, education, social support, language, and health literacy. However, a study by Enard et al. examined 1373 articles and found that social determinants of health were inconsistent and unreliable when used to predict HF [12]. The inconsistency can be attributed to the different definitions and methodologies used to measure social determinants of health across the studies [12]. This is not to claim that social determinants of health have no influence on HF, but rather, it underlines the importance of a standardized and replicable method to effectively report and collect data across studies. It has been observed that social determinants of health, such as access to reliable transportation, availability of grocery stores with healthy food options, stable and safe housing, and well-funded schools, are all factors that may affect the ability of an individual to seek appropriate healthcare, adhere to treatment plans, and make informed decisions about their health [13]. These factors play an important role in shaping healthy behavior, and it is crucial to address them to reduce disparity in healthcare. The battle against structural racism has been ongoing in the USA, and consistent findings will help to target the specific social determinants of health to reduce HF in Black communities.

Hospitalization and readmission rates remain a pressing concern among Black populations with HF. This demographic accounts for nearly 50% of HF admissions among adults aged 18 to 45. Multiple factors, including limited healthcare access, socioeconomic barriers, and inadequate follow-up care, contribute to the elevated hospitalization and readmission rates [14]. Notably, the COVID-19 pandemic further highlighted these disparities, with Black individuals experiencing increased admissions and complications due to exacerbations of preexisting heart conditions. Addressing these disparities requires a comprehensive approach that improves healthcare access, enhances continuity of care, and targets the underlying social determinants impacting health outcomes [15].

HF remains a significant health challenge globally, with Black populations in the USA experiencing a higher prevalence, earlier onset, and greater severity compared to other racial and ethnic groups [16]. Recent studies have shown that these disparities are not only persistent but may have been exacerbated by the COVID-19 pandemic [17]. Black populations were disproportionately affected by COVID-19, with higher rates of infection, hospitalization, and mortality due to underlying health disparities and preexisting conditions like HF. The pandemic worsened symptoms, increased hospitalization rates, and complicated the management of comorbidities such as hypertension and diabetes. These factors, along with disruptions in healthcare services, underscore the need for targeted interventions that address both clinical and social determinants of health [18].

## Pathophysiological Factors

Black individuals may exhibit distinct pathophysiological characteristics that influence their presentation and vulnerability to HF. HF has been linked to hypertrophic cardiomyopathy or dilated cardiomyopathy. In this regard, mutations of several genes also have been implicated [19]. When compared to White individuals, Black individuals have a threefold higher risk of developing dilated cardiomyopathy, a phenomenon not solely explained by socioeconomic factors [20, 21]. Research conducted on a Black population through genome sequencing revealed a previously unidentified intronic site within the Calcium Voltage-Gated Channel Auxiliary Subunit Beta 4 (CACNB4) gene, crucial for skeletal muscle contraction due to its role in calcium channel subunit regulation [14, 15]. Another study focusing on Black patients with dilated cardiomyopathy identified four specific genetic variations in Bcl2-associated anthanogene 3 (BAG3), exclusive to this demography, correlating with a two-fold increase in the risk of death or hospitalization due to HF [22]. A multinational study involving women with peripartum cardiomyopathy found a prevalence of truncating variants among Black women, possibly elucidating the more severe disease progression, left ventricular dysfunction, and diminished left ventricular recovery observed in this group compared to their White counterparts [22]. Identifying the genetic factors responsible for HF in Black individuals is important for developing therapeutics and treatment plans to target the unique pathophysiological factors involved.

In addition, numerous more genetic factors have been discovered to play a role in the increased risk of HF among Black individuals. For example, transthyretin-related cardiac amyloidosis, which is attributed to a valine-to-isoleucine substitution at position 122 on chromosome 18, carries a significant risk for HF, affecting 3–5% of Black individuals [23]. A landmark study conducted in Los Angeles County revealed that the prevalence of cardiac amyloidosis was significantly higher among Black individuals (1.6%) compared to White Americans (0.42%), highlighting a potential genetic basis for this disparity [24]. This condition is largely attributed to a mutation in the transthyretin (TTR) gene, which is predominantly found in individuals of Black descent. Studies indicate that 3–4% of self-identified Black patients carry this TTR gene variant (V142I), whereas it is rare among other ethnic groups. The highest prevalence of this variant is reported in the West Black population, where approximately 5% of the population carries the allele [25]. Thus, Black patients should receive appropriate diagnostic testing to test for TTR.

Furthermore, the renin–angiotensin–aldosterone system (RAAS) is a crucial hormonal system that helps regulate blood pressure, fluid balance, and electrolyte levels [23]. A study showed that a common polymorphism exists in the promoter region of the aldosterone synthase gene (CYP11B2) at position − 344 (T/C). The − 344C allele, associated with higher aldosterone synthase activity, binds to a steroidogenic transcription factor 1 four times more than the T-allele and is thus linked to hypertension [23]. This polymorphism is more prevalent in Black patients and is associated with poorer HF outcomes.

Another important gene that affects the prevalence of HF in Black patients is mutations in MYH7, which encodes the major myosin heavy chain (MHC) in the heart. Mutation of this gene was implicated in the causation of hypertrophic cardiomyopathy (HCM) [19]. In healthy individuals, myosin is a protein responsible for cardiac contraction. Expression of different myosin isoforms can be linked with normal aging; however, it was also observed that more variations in MYH7 have been linked with impaired contractility and familial cardiomyopathy [26]. Black populations express significantly more variations in MYH7 than all other ethnic groups in the USA [27]. It might be beneficial for future studies to explore the benefits of increased screening procedures to assess cardiac contractility in individuals with family history and risk for MYH7 mutations.

While genetic variations like the TTR mutation increase susceptibility to cardiac amyloidosis and HF in Black patients, there are also significant differences in the biochemical pathways involved in HF. One such pathway involves natriuretic peptides, which play a critical role in maintaining cardiovascular homeostasis.

Natriuretic peptides (NPs) are generated in response to heightened tension and stress in the cardiac walls, offering several protective cardiometabolic benefits such as vasodilation, and encouragement of natriuresis and diuresis [28]. Research in animals has shown that compromised NP production contributes to the development of salt-sensitive hypertension and cardiac hypertrophy [29]. Black patients have a deficiency in the production of NP, which increases their predisposition to various heart conditions. Ten to twenty percent of the Black population has been shown to possess a genetic variant of the CORIN gene, a serine protease that normally activates NP in the cardiomyocyte [22, 23, 30]. In a study that analyzed gender and race differences in cardiac structure, Black patients were more likely to have a lower mean left ejection fraction [28]. Even after adjusting for cardiac comorbidities, Black patients were also associated with greater left ventricular wall thickness and concentricity. In older patients without HF, HF patients demonstrated worse systolic performance. In conclusion, it is necessary to understand the genetic factors and differences in pathophysiology contributing to HF to develop tailored treatment protocols and screenings.

Furthermore, it is crucial to understand the phenotypes of H that disproportionately affect patients, particularly HF with preserved ejection fraction (HFpEF). Black populations have been observed to exhibit higher rates of HFpEF, which is commonly associated with comorbid conditions like obesity and hypertension. This phenotype presents unique challenges in management due to limited treatment options and its association with poorer outcomes compared to HF with reduced ejection fraction (rEF) [21]. Tailoring treatment approaches to effectively address these phenotypes is essential in reducing morbidity and improving the long-term prognosis of HF in this population.

## Treatment Disparities and Outcomes

Black individuals with HF may have a reduced tendency to access healthcare services, leading to inferior health outcomes. In a recent group of patients admitted to the intensive care unit (ICU) with HF, Black individuals were less frequently admitted by a cardiologist compared to Caucasians [31]. However, regardless of race, being admitted by a cardiologist rather than a non-cardiologist was linked to improved in-hospital survival rates [32]. In addition, low socioeconomic status may cause a lack of adherence to treatment plans due to an inability to access or afford medications. The *Association of Black Cardiologists* highlights several significant barriers that minorities, including Black patients, face in accessing adequate healthcare. These obstacles include lack of health insurance, limited proximity to healthcare providers, language, and cultural barriers, as well as low levels of health literacy. These factors collectively contribute to disparities in healthcare access and outcomes among minority populations [33].

It is essential to acknowledge the heterogeneity within Black populations, as various subgroups may experience different barriers in healthcare. While language and cultural barriers are commonly reported, the context varies significantly between immigrant and American-born Black individuals. Immigrant populations may face language challenges and cultural misunderstandings, whereas American-born Black individuals might experience implicit biases and communication gaps [34]. Furthermore, patient-provider discordance, characterized by differences in cultural beliefs, communication styles, and trust in the healthcare system, can also contribute to disparities in HF management [35]. These factors underscore the complexity of healthcare experiences within the Black community, necessitating a nuanced approach to addressing disparities.

Black populations suffer from a high prevalence and incidence of HF, but they are also regularly under-prescribed HF guideline-directed medical therapy (GDMT). GDMT refers to specific disease management using the most up-to-date clinical evidence and guidelines set by the major medical organizations [36].

In recent years, updates to guideline-directed medical therapy (GDMT) for HF have emphasized optimizing care for patients with HF with reduced ejection fraction (rEF) and HF with preserved ejection fraction (pEF). According to the 2024 ACC Expert Consensus, the standard GDMT now includes comprehensive use of ACE inhibitors or ARBs, beta-blockers, mineralocorticoid receptor antagonists (MRAs), and sodium-glucose co-transporter-2 (SGLT2) inhibitors for HF rEF [37]. For Black patients, the guidelines continue to advocate the use of the hydralazine-isosorbide dinitrate combination due to its proven benefits in reducing morbidity and mortality. The 2024 updates also stress the importance of early initiation of SGLT2 inhibitors and guideline-directed optimization in patients at higher risk of adverse outcomes, particularly those with comorbidities like hypertension and diabetes, which disproportionately affect Black populations. Integrating these updated recommendations is crucial for reducing disparities in HF care.

The addition of isosorbide dinitrate in combination with the standard dose of care hydralazine resulted in elevated survival rates and decreased hospitalization rates among Black patients with HF [38]. A European study by Brewster et al. (2019) found that the combination of hydralazine and isosorbide nitrate for HF was generally under-prescribed for Black patients [39]. In a study involving 54,622 patients with HF, researchers assessed the use of hydralazine-isosorbide dinitrate, a treatment specifically beneficial for Black patients. Among the eligible Black patients, only 22.4% received this treatment, highlighting a significant underutilization despite established benefits for this population. In contrast, across the entire study cohort, only 12.6% of all patients received this combination therapy. These findings underscore disparities in the prescription of guideline-directed medical therapy, particularly for Black patients, where issues such as lack of insurance were the most common barriers to accessing optimal care [40]. The combination of hydralazine-isosorbide dinitrate has been proven to be beneficial for Black patients, yet studies reveal that its therapy is often under-prescribed in these HF patients. Addressing this lack of prescription and ensuring that these patients receive GDMT specific to their needs are crucial in improving healthcare outcomes. Cost and the lack of insurance should not be justification for deviating from GDMT.

In 2008, the OPTIMIZE-HF study sought to analyze the quality of care and clinical outcomes for HF regarding Black patients compared to non-Black patients. The study took place in the USA and included 48,612 patients. The study findings revealed that Black patients received equivalent or superior care in some respects, such as testing for left ventricular function and prescriptions of angiotensin-converting enzyme inhibitors. However, they also found that Black patients were less likely to receive clear discharge instructions and counseling on tobacco use [41]. The equivalence in quality of care is promising, but the lack of discharge counseling needs to be addressed.

In a study by Witting et al. (2022), researchers analyzed GDMT disparities in a group of 126,670 patients with HF within the Veterans Affairs (VA) healthcare system [42]. The results showed that ethnic minorities received similar or increased treatment rates when compared to White patients. When location was considered, various differences in care and management were observed. For example, socially vulnerable neighborhoods received lower treatment rates with angiotensin receptor blocker-neprilysin inhibitors treatment. Furthermore, patients living in remote areas were less likely to have their beta-blocker and renin-angiotensin converting enzyme inhibitor doses increased to the target range compared to those living closer to specialty care. Proposed solutions for patients living in remote areas include the increasingly popular use of telemedicine, home health, and mail-in-prescriptions [42].

Exercise training and cardiac rehabilitation (CR) are safe and effective adjunctive therapies associated with improved quality of life and decreased HF mortality and hospitalizations [32–34]. US and European guidelines provide Class I recommendations for stable HF patients to include exercise training in standard HF management [35, 36]. Though cardiac rehabilitation is generally underutilized in the overall population, racial and ethnic minorities are significantly less likely to receive cardiac rehabilitation referrals [43]. In light of the mortality and functional benefits, significant attention should be focused on eliminating barriers to referral, enrollment, availability, and completion of CR [38–40].

## Future Directions

The care of patients with HF has improved drastically in the last decade through concern about physical exercise and a healthy diet, understanding the symptoms, and a combination of pharmacological innovations, device-based interventions, and regenerative medicine that could offer synergistic effects in managing HF*.* However, there persist important disparities among Black patients [44]. It was reported that *Black* men and women experience a rate of death associated with HF 2.6 and 2.97 folds higher, respectively, when compared to non-Hispanic White men and women [21]. This burden is not only associated with an increased mortality rate, but Black individuals also spend nearly 2.5-fold more compared to non-Hispanic White counterparts due to increased rates of hospitalizations and complications [1]. The published data suggest that the strongest associated factors are socioeconomic conditions such as income, wealth, and education, which enhance access to care and alleviate suffering; however, there is also a strong association with genetic susceptibility that has a contributing role in creating this disparity [45]. The prevalence of cardiovascular risks, such as hypertension, obesity, and chronic kidney disease, is much higher in Black individuals compared to other race-ethnic groups [46]. This indicates that a higher prevalence of modifiable cardiovascular risk factors is the main contributing factor to the increased prevalence of HF among the Black population [18].

The reduction of this disparity can be achieved by increase by focusing on the social determinants that are lacking in Black communities. The strongest determinant is socioeconomic status. Socioeconomic status was shown to be an indicator of stability, housing, access to care, and lower risk of CVD (cardiovascular disease) events such as HF [47]. Decades after the abolishment of slavery and Jim Crow laws, people of color are still discriminated against in employment opportunities, leading to unstable socioeconomic conditions [46]. Future research is needed to investigate the consequences of low income on HF over time, assessing the levels of stress associated with economic instability, poor diet due to reduced affordability, and strenuous work environments that are potentially contributing to the development of the comorbidities. It has already been documented that disadvantaged neighborhoods and unsafe physical environments have been shown to be associated with an increased risk of HF and worsened CV outcomes [9]. The insecurity of an individual’s environment has been shown to increase risks of insulin resistance, hypertension, and metabolic disorders. In addition, some areas are associated with restricted healthcare access, thereby reducing the ability of patients to participate in preventative measures and adequate health assessments [46]. There needs to be widespread attention and efforts to improve these environmental conditions, creating environmental spaces that are safe and secure and ensuring adequate healthcare access to under-served areas. While specific interventions are ongoing, several strategies have been implemented to improve cardiovascular health outcomes: (1) environmental improvements which reduce exposure to environmental hazards. Efforts have been made to address environmental justice issues, such as those in Louisiana’s “Cancer Alley,” where predominantly Black communities face high pollution levels linked to industrial facilities [48]. Advocacy and policy changes aim to reduce exposure to pollutants that contribute to cardiovascular diseases. (2) Community safety enhancements indicate that unsafe neighborhoods can limit physical activity; initiatives have focused on enhancing community safety. This includes better lighting, community policing, and creating safe public spaces to encourage exercise, which is vital for heart health [49]. (3) Improving housing and reducing poverty which have been implemented, recognizing their impact on health outcomes. For example, addressing substandard housing can reduce stress and exposure to environmental hazards, thereby improving health [50]. (4) Community-based health programs have been established to provide education on heart health, conduct screenings for hypertension and other risk factors, and promote healthy lifestyles. These programs often partner with local organizations to reach Black communities effectively [51]. (5) Promoting health equity research focusing on health disparities has increased, leading to a better understanding of the unique challenges faced by Black populations regarding HF. This research informs policy and healthcare practices aimed at reducing these disparities [52]. While all these efforts are steps towards improving heart health in Black communities, ongoing commitment and comprehensive strategies are essential to address the multifaceted factors contributing to HF disparities. In addition, the educational challenge for under-represented communities is also acting as a barrier to healthy living, food security, and healthcare [46].

The healthcare system should take up the role of reshaping the landscape for racial justice. It was reported in 2018 that nearly 7.3 million nonelderly adults with HF were uninsured [46]. Despite improvement efforts, like the Affordable Care Act, there is a huge disparity in uninsured rates among Hispanics and Black individuals [53]. Efforts include improving health and education, access to affordable healthcare for the underserved population, food security, heart-healthy diets, limiting alcohol, implementing culturally tailored smoking cessation programs, and promoting community-based exercise initiatives. Hospitals located in underserved areas have been shown to have higher readmission rates from HF, which is mainly attributed to poor quality of care [54]. Increased resources and assistance in hospitals in underserved areas is a solution that can reduce readmission rates and potential mortality among Black patients with HF. To effectively reduce the significant disparities in HF outcomes among Black patients, it is essential to address the underlying social determinants of health, particularly socioeconomic status, through comprehensive strategies that include improving healthcare access, enhancing community safety, increasing educational opportunities, and ensuring equitable treatment within the healthcare system [46].

## Conclusion

In navigating the multifaceted landscape of HF among Black patients, this review underscores the critical imperative for targeted interventions and a deeper understanding of the underlying factors perpetuating disparities. As HF continues to exert a significant toll on individuals, families, and healthcare systems globally, the poignant narrative of inequity among Black populations remains a pressing concern. The disparities in HF prevalence, treatment, and outcomes among Blacks underscore the urgent need for tailored interventions and comprehensive strategies aimed at mitigating these inequities. Despite advancements in medical science and healthcare delivery, Black patients endure a higher burden of HF, marked by earlier onset and increased severity compared to other racial and ethnic groups.

From examining the epidemiological patterns to unraveling the intricate pathophysiological mechanisms and exploring treatment disparities, this review illuminates the unique challenges faced by Black patients with HF. By synthesizing existing literature and advocating for evidence-based interventions, we strive to foster meaningful dialogue and actionable initiatives to enhance the well-being of vulnerable populations. Addressing social determinants of health, enhancing access to care, and promoting health equity are paramount in reducing disparities in HF outcomes among Black patients. As we navigate these challenges, it is imperative to adopt a holistic approach that addresses the systemic barriers and socio-economic determinants perpetuating disparities in HF care and outcomes.

This review aims to not only develop a comprehensive understanding of HF in Black but also catalyze efforts towards equitable care and improved outcomes for all individuals affected by this debilitating condition. By advocating for a more inclusive and equitable healthcare system, we can strive towards a future where disparities in HF outcomes are minimized, and all individuals have access to the care they need to live healthier lives.
